# Hypolipidemic activities of partially deacetylated α-chitin nanofibers/nanowhiskers in mice

**DOI:** 10.29219/fnr.v62.1295

**Published:** 2018-07-17

**Authors:** Wenbo Ye, Liang Liu, Juan Yu, Shilin Liu, Qiang Yong, Yimin Fan

**Affiliations:** 1 Jiangsu Co-Innovation Center of Efficient Processing and Utilization of Forest Resources, Jiangsu Key Lab of Biomass-Based Green Fuel and Chemicals, College of Chemical Engineering, Nanjing Forestry University, Nanjing, China; 2 College of Food Science and Technology, Huazhong Agricultural University, Wuhan, China

**Keywords:** chitin, nanofibers/nanowhiskers, hypolipidemic effects, cholesterol

## Abstract

Partially deacetylated α-chitin nanofibers/nanowhiskers mixtures (DEChNs) were prepared by 35% sodium hydroxide (NaOH) treatment followed by disintegration in water at pH 3–4. The aim of this study was to investigate the hypolipidemic effects of DEChNs at different dosage levels in male Kunming mice. The male mice were randomly separated into five groups, that is, a normal diet group, a high-fat diet group, and three DEChN groups that were treated with different doses of DEChN dispersions (L: low dose, M: medium dose, H: high dose). Primarily, the DEChNs significantly decreased body weight (BW) gain and adipose tissue weight (ATW) gain of mice. Meanwhile, the decreasing extent of weight ratios between ATW and BW was dependent on the dose of DEChNs. Moreover, the DEChNs prevented an increase in plasma lipids (cholesterol and triacylglycerol) in mice when they were fed a high-fat diet. Histopathological examination of hepatocytes revealed that the DEChNs were effective in decreasing the accumulation of lipids in the liver and preventing the development of a fatty liver. The results suggested that the DEChNs reduced the absorption of dietary fat and cholesterol *in vivo* and could effectively reduce hypercholesterolemia in mice.

Hyperlipidemia is a major cause of coronary atherosclerosis and subsequent related cardiovascular disease, which has always been related to obesity to some extent (1). In recent years, most studies have examined how to decrease plasma lipid concentrations and the absorption of fat in the intestinal tract to reduce diet-related chronic disease (2). The conventional therapeutic modalities for hyperlipidemia are lipid-lowering drugs such as atorvastatin, lovastatin and fibrates (3). These synthetic drugs are available and effective but may cause adverse effects at the same time (4). Therefore, the antihyperlipidemic activity of many biologically active components from natural materials, such as polysaccharides and dietary fiber, has been explored. Polysaccharides, such as *Ulva pertusa*, *Ulva lactuca*, chitosan, and chitosan derivatives, are novel potential hyperlipidemic agents (3). Other dietary fibers, such as pectin and psyllium, also exhibit potent hypolipidemic effects (5).

Studies have shown that chitosan has a beneficial lowering effect on plasma cholesterol, which may play an important role in the prevention and treatment of cardiovascular disease (6, 7). Some previous research work has revealed the hypolipidemic mechanism of chitosan hypolipidemic activity of low molecular weight chitosan was better than high molecular weight chitosan (8, 9). The positively charged amino groups of chitosan may have the ability to bind negatively charged molecules, such as lipids and bile acids, and subsequently be excreted in the feces (10). The adsorption of chitosan to oil droplets affects the digestibility of oil. Access to the oil by digestive enzymes is decreased, thereby decreasing oil digestion.

Chitosan is a derivative of chitin. Chitosan is a cationic polysaccharide produced by the deacetylation of chitin under alkaline conditions, normally with a degree of deacetylation (DDA) greater than 0.7. Chitosan is, consequently, a copolymer of N-acetyl-D-glucosamine and D-glucosamine, which is similar to chitin structurally (11). Chitin, the source of chitosan, is the second most abundant renewable natural polysaccharide after cellulose. Chitin is the main component of the exoskeleton of arthropods, such as crabs, prawns, and shrimp, as well as the cuticles of insects and the cell walls of certain fungi, coexisting with various types of proteins and certain minerals (12). Isolated chitin is composed of β-(1-4)-linked 2-acetamido-2-deoxy-β-D-glucose units and a small amount of D-glucosamine. Because a large amount of crab and shrimp shell–derived chitins is discarded every year, utilizing chitin as a functional material or commodity is highly beneficial (13). In addition to converting chitin to its highly deacetylated derivative, chitosan, which may be used in a variety of applications, chitin itself has the potential to be converted into individual nanofibers through some downsizing processes.

One of the methods, partial deacetylation with 30–35 wt% NaOH treatment followed by disintegration in water at a pH of 3–4, was used to prepare partially deacetylated α-chitin nanofiber/nanowhisker mixtures (DEChNs) from α-chitins. The as-prepared DEChNs had DDA values of 0.25–0.35 and a high density of cationic charged C2-amino groups on the surface (14). The DEChNs had lower DDA values (0.25–0.35) than chitosan (normally >0.70) and did not dissolve at a molecular level but dispersed at a nanofibril level, maintaining high crystallinity (14). Our previous studies showed that partially deacetylated α-chitin can be converted to nanofiber/nanowhisker mixtures by mechanical pretreatment under acidic conditions (15). The DEChNs have been studied as nanostructured materials for further applications, such as gels and films (16). Other research work has been conducted to study the biochemical properties of DEChNs. Kazuo Azuma et al. evaluated the effects of oral administration of surface-deacetylated chitin nanofibers, chitosan, and cellulose nanofibers on hypercholesterolemia in rats and indicated that chitin nanofibers suppressed an increase in serum total cholesterol (TC), chylomicron, very-low-density lipoprotein (VLDL), and phospholipid (PL) levels while increasing alanine transaminase levels, vacuolar degeneration, and accumulation of lipid droplets in liver tissue (17). Recently, Anraku et al. proposed that surface-deacetylated chitin nanofibers are more effective in decreasing renal injury and oxidative stress than deacetylated chitin powder in 5/6 nephrectomized rats (18). The result of this study suggested that ingestion of chitin nanofibers, not chitin powder, resulted in significantly reduced levels of pro-oxidants, such as uremic toxins, in the gastrointestinal tract, thereby inhibiting the subsequent development of oxidative stress in the systemic circulation. All of these studies stated the potential of chitin in the biotechnology field, emphasizing the importance of nanofibrillation of chitin. However, research has rarely reported the mechanism and the effects of chitin nanofibers/nanowhiskers on hypolipidemic function. Although the DEChNs and chitosan have similar macromolecular structures, DEChNs are characterized by nanofibril morphology, lower amino groups, and higher crystallinity. Thus, it is worth further examination of the relationship between the biochemical properties of DEChNs and their hypolipidemic effects.

In the present study, the hypolipidemic effects of the DEChN dispersions were investigated in male Kunming mice at different dosage levels. The effects of the DEChNs on the weight of the body, organ tissue, and adipose tissue were examined in this study. Serum blood chemistry analysis and histopathological examination of the liver were also performed.

## Materials and methods

### Materials

The α-chitin was purified from swimming crab (*Portunus trituberculatus*) shells collected from Nantong, a seaside city in Jiangsu Province, China, as follows (11). Discarded crab shells were soaked in 1 M HCl for 12 h to remove the mineral salts. The sample was then washed thoroughly with distilled water, followed by treatment with 1 M NaOH for 12 h to remove the proteins. These two steps were repeated three times. The obtained residual solid was decolored by immersing it in 0.5% (w/w) NaClO_2_, and the pH was adjusted to 5 using acetic acid. The suspension was heated for 2 h at 70°C. The purified α-chitin solid residues were obtained by filtration, rinsed with distilled water, and stored at 4°C for further use. TC, triacylglycerol (TG), high-density lipoprotein cholesterol (HDL-C), and low density lipoprotein cholesterol (LDL-C) kits were purchased from Nanjing Jiancheng Bioengineering Institute, Nanjing, China. All other reagents and solvents were of analytical grade.

### Preparation of deacetylated a-chitin nanofiber/nanowhisker mixtures

The α-chitin (1 g) was suspended in 35% (w/w) NaOH solution (25 mL) at 90°C for 4 h with continuous stirring. The final partially deacetylated chitin product with a DDA of 0.29 was collected and thoroughly washed with deionized water until a neutral pH was obtained and then stored at 4°C (14). The DDA of the original and partially deacetylated chitins were determined by the electrical conductivity titration method (19). In this case, a dried sample (0.1 g) was added to water (60 mL), and a small amount of 0.5 M NaOH was used to adjust the pH to 9. The slurry was stirred for 30 min for adequate distribution. Afterward, 0.1 M HCl was added to adjust the pH to 2.5–3.0, and a 0.05 M NaOH solution was added at a rate of 0.1 mL/min to adjust the pH to 11 using a pH-stat titration system. The conductivity and pH curves obtained reflect the amount of C2-amino groups in the chitins.

The partially deacetylated chitin was suspended in deionized water at a concentration of 0.1% (w/v), and the pH of the suspension was adjusted from the original pH of 6–7 to 3–4 by adding acetic acid with constant stirring. Next, this suspension was homogenized and treated with ultrasonication, the DEChNs were obtained after centrifugation, and the dispersions were successfully prepared (16).

### Animals and diets

Kunming mice (males, Specific pathogen free (SPF), 18–20 g) were used as the experimental animals. The mice were procured from the Animal Center for Disease Prevention and Control in Hubei Province, China. All animal protocols were approved by the institutional animal care and use hospital of Huazhong Agricultural University (Wuhan, China). The animal study was performed under strict adherence to the international rules for animal experiments and the internationally accepted ethical principles for laboratory animal use and care. The mice were housed in cages in a controlled environment (25 ± 2°C, 50–70% relative humidity, 12±1 h light–dark cycle). There were five mice in each cage, and these mice were allowed free access to food and water for 5 days. Then, the mice were randomly divided into five groups as follows (10 mice per group). The normal fat control group (blank group, *n* = 10) received a normal fat diet and saline solution. The normal fat diet consisted of 40% corn starch, 22% wheat, 20% soybean, 7% peptone (fish), 4% casein, 4.2% mineral mixture, 0.8% amino acid, 0.3% vitamin mixture, 0.2% choline chloride, and 1.5% fat. The high-fat control group (control group, *n* = 10) received a high-fat diet and dilute acetic acid solution. Finally, there were three DEChN groups, namely, the L-DEChN (*n* = 10), M-DEChN (*n* = 10), and H-DEChN groups (*n* = 10), which received a high-fat diet and low, medium, or high doses of DEChN dispersions, respectively, dispersed in dilute acetic acid at a pH of 3–4. The high-fat diet consisted of 10% lard, 10% egg yolk powder, 2% cholesterol, 0.5% sodium deoxycholate, and 77.5% normal fat diet.

### Experimental design

All mice were fed under experimental conditions for 21 days. The male mice were randomly separated into five groups: a normal diet group, a high-fat diet group, and three DEChN groups that had been treated with different doses of DEChN dispersions (low, medium, and high). During the experimental period, the method described by Qi et al. (20) was employed in this study. The mice were allowed free access to food and water for 5 days to acclimate to the animal facility and were weighed and randomly divided into five groups as described above. Each day the body weights (BW) were recorded, and in the DEChN groups, the mice were intragastrically administered DEChN dispersions at dosages of 18.75 mg/kg·bw·d (L-DEChN group), 37.5 mg/kg·bw·d (M-DEChN group), and 75 mg/kg·bw·d (H-DEChN group). Correspondingly, each day the mice in the blank group were given saline solution, and the mice in the control group were given dilute acetic acid solution. At the end of the third week, mice fasted for 18 h prior to blood withdrawal. Necropsies were performed on all mice after blood withdrawal, and organ and adipose tissues were collected and measured.

### Collection of blood and bioassay

All blood samples for the bioassay were obtained by retro-orbital blood collection from mice that fasted overnight (18 h). Blood samples were centrifuged at 3,000 g for 10 min to separate the serum for lipid profile estimation and stored at –4°C. The serum TC, TG, HDL-C, and LDL-C concentrations were measured by an enzymatic colorimetric method using commercially available kits. Meanwhile, liver, kidney, and spleen samples were also collected and weighed.

### Statistical analysis

Statistical analyses were performed using Student’s *t*-test and compared to the control group (21). The data are presented as the mean ± standard deviation; *p* < 0.05 was considered a statistically significant difference.

## Results and discussion

### Characterizations of deacetylated a-chitin nanofiber/nanowhisker mixtures

**Fig. 1:**
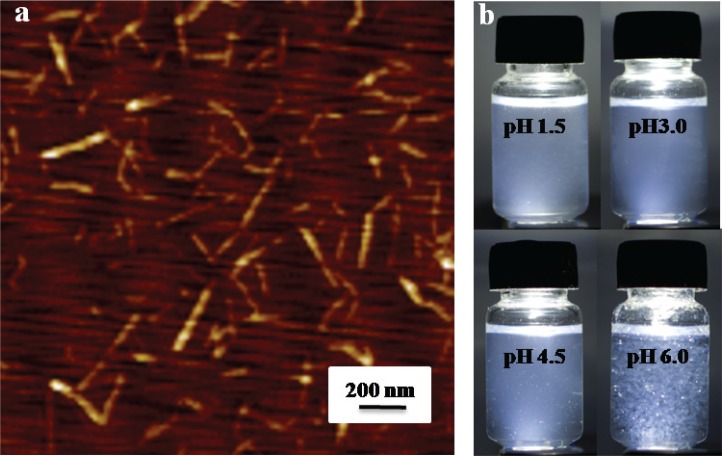
Characterizations of partially deacetylated α-chitin nanofibers: (a) atomic force microscopy observation of partially deacetylated α-chitin nanofiber/nanowhisker mixtures (DEChNs) prepared from α-chitin; (b) photographs of dispersions of DEChNs in water at pH 1.5–6.

The atomic force microscopy image of the DEChNs is shown in Fig. 1a. Complete individualization of DEChNs was achieved by partial deacetylation and the following downsizing process. The as-prepared individual DEChNs had widths that ranged consistently between 10 and 30 nm and lengths that ranged between 150 and 500 nm. The DDA of the DEChNs was 0.29.

Photographs of the DEChNs in water at pH 1.5–6 are shown in Fig. 1b. The DEChNs were well dispersed in water at pH 1.5–4.5; however, at pH 6.0 there was severe turbidity with white flocculent particles, indicating that DEChNs flocculate in water at higher pH values. The well dispersion of DEChNs at pH 1.5–4.5 was in accordance with its high zeta-potential (+30–70 mV), which indicated the high cationic surface charges of DEChNs. Thus, the addition of acetic acid cationized the C2-amino groups on the DEChNs, promoting the fibrillation of the DEChNs into nanofibrils by electrostatic repulsion and stable dispersion in water. However, DEChNs lost a certain amount of positive charge when the zeta-potential decreased to +24.1 mV at pH 6.0 and therefore became flocculent and precipitated.

While pH is normally neutral in most parts of the body, it is acidic in the gastric environment. The changes in the zeta-potential and dispersion-flocculation that occurred in relation to the pH value of the DEChNs reflect the potential of DEChNs to bind molecules such as lipids, cholesterol, and bile acid in the stomach at pH < 4.5 (acidic gastric juice) and to flocculate with the binding molecules at a higher pH in the intestine for excretion outside of the body.

### Body and tissue weight of mice

**Fig. 2:**
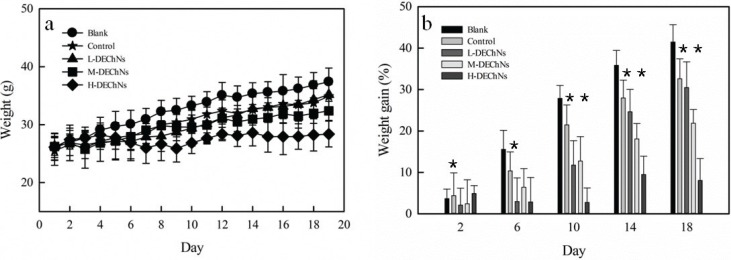
(a) Changes in body weight and (b) the difference in body weight gain in the blank group (fed a normal diet and saline solution), control group (fed a high-fat diet and dilute acetic acid solution), and L-, M-, and H-DEChN groups (fed a high-fat diet and low, medium, and high doses of DEChN dispersions). ^*^p < 0.05 control group compared with the DEChN groups. ^**^p < 0.01 control group compared with the DEChN groups.

The BW and BW gain of experimental mice are shown in Fig. 2a and b, respectively. The DEChN dispersions were divided into the following three dosage groups: L-DEChNs, M-DEChNs, and H-DEChNs, in which the mice were fed dosages of 18.75, 37.5, and 75 mg/kg·bw·d of DEChN dispersions, respectively. The mice were fed the DEChNs along with a high-fat diet to estimate their effect on the resulting weight change of the mice. As a control, because the DEChNs were dispersed under acidic conditions mediated by acetic acid, the dilute acetic acid solution was given to the mice along with the high-fat diet.

As shown in Fig. 2a and b, the mice fed the high-fat diet plus the dilute acetic acid solution (control group) showed decreases in BW during the experimental period, compared with those mice fed the normal-fat diet plus a saline solution (blank group). This indicates that the dilute acetic acid could reduce the weight gain of the mice even though they were fed the high-fat diet. Until Day 14, there were some differences in the BW of mice fed the L-DEChNs and mice in the control group. However, after 14 days, the BW of these two groups showed little difference. The results suggested that the L-DEChN (18.75 mg/kg·bw·d) dispersion dosage slightly mitigated increases in the BW of mice fed a high-fat diet after the initial 2 weeks, when mice had acclimated to the feeds. Compared with the control group and the L-DEChN group, there was significantly less BW gain in mice that had been fed the M-DEChN and H-DEChN doses. Especially for the mice that received H-DEChNs along with a high-fat diet, a minimal increase in the BW was observed.

Overall, the BW of the mice obviously changed; both acetic acid and DEChNs were able to prevent the weight gain of mice, even those fed a high-fat diet, and the degree of prevention was correlated with the dosage of DEChNs.

Necropsies were performed on the experimental mice after 21 days. The tissue weights (TW) of the mice (including liver weight, kidney weight, and spleen weight) are shown in Table 1. As shown in Table 1, compared with the control group, the TW of the mice fed DEChN dispersions were significant lower along with a lower overall BW. The results suggest that DEChNs reduced both the BW and TW gain of mice. In contrast, the ratios of liver, spleen, and kidney weights to BW (TW/BW) showed no significant differences among all groups.

**Table 1:** Body and tissue weights of mice

Groups	Blank^a^	Control^b^	L-DEChNs^c^	M-DEChNs^d^	H-DEChNs^e^
BW (g)	33.63 ± 2.87	34.19 ± 1.89	31.88 ± 3.02^*^	30.36 ± 3.26^**^	26.58 ± 2.08^**^
Liver weight (g)	1.92 ± 0.18	1.87 ± 0.30	1.67 ± 0.15^*^	1.64 ± 0.14^*^	1.46 ± 0.04^**^
Liver weight/BW (%)	5.70 ± 0.30	5.48 ± 0.55	5.23 ± 0.31	5.40 ± 0.34	5.51 ± 0.21
Spleen weight (g)	0.116 ± 0.035^*^	0.139 ± 0.016	0.133 ± 0.022	0.1195 ± 0.03	0.113 ± 0.046
Spleen weight/BW (%)	0.34 ± 0.08^*^	0.41 ± 0.04	0.42 ± 0.08^*^	0.39 ± 0.05^*^	0.43 ± 0.06
Kidney weight (g)	0.672 ± 0.124	0.681 ± 0.09	0.637 ± 0.067	0.571 ± 0.131^*^	0.497 ± 0.024^*^
Kidney weight/BW (%)	1.996 ± 0.345	1.99 ± 0.14	1.999 ± 0.141	1.88 ± 0.33	1.87 ± 0.16

BW, body weight

a Blank group (fed normal diet and saline solution);

b control group (fed high-fat diet and dilute acetic acid solution);

c,d,e L-, M-, H-DEChN groups (fed high-fat diet and low, medium, high dose, respectively, of DEChN dispersions). Data are presented as mean ± standard error.

* *p* < 0.05 compared with the control group;

** *p* < 0.01 compared with the control group.

### Adipose tissue and serum lipids

The adipose tissues (including renal, epididymal, and mesenteric adipose tissue) of experimental mice were collected and measured. The adipose tissue weight (ATW) and the ATW/BW ratios are shown in Table 2. Compared with the mice fed the normal-fat diet plus a saline solution (blank group), the mice fed the high-fat diet plus a dilute acetic acid solution (control group) showed an increase in ATW at the end of the experimental period. This indicates that feeding the mice a high-fat diet resulted in a high accumulation of lipids.

**Table 2:** Adipose tissue weight of mice

Groups	Blank^a^	Control^b^	L-DEChNs^c^	M-DEChNs^d^	H-DEChNs^e^
Renal ATW (g)	0.234 ± 0.116^*^	0.418 ± 0.148	0.235 ± 0.068^*^	0.268 ± 0.075^*^	0.146 ± 0.041^**^
Renal ATW/BW (%)	0.69 ± 0.30^**^	1.21 ± 0.37	0.72 ± 0.21^*^	0.88 ± 0.19^*^	0.54 ± 0.11^**^
Epididymal ATW (g)	0.695 ± 0.107	0.819 ± 0.238	0.621 ± 0.16	0.700 ± 0.159	0.412 ± 0.098^*^
Epididymal ATW/BW (%)	2.06 ± 0.27	2.38 ± 0.72	1.95 ± 0.47	2.20 ± 0.22	1.54 ± 0.23^*^
Mesenteric ATW (g)	0.616 ± 0.17	0.728 ± 0.085	0.476 ± 0.187	0.594 ± 0.115^*^	0.471 ± 0.097^*^
Mesenteric ATW/BW (%)	1.83 ± 0.35^*^	2.13 ± 0.61	1.48 ± 0.40^*^	1.96 ± 0.38^*^	1.75 ± 0.31

ATW, adipose tissue weight; BW, body weight

a Blank group (fed a normal diet and saline solution);

b control group (fed a high-fat diet and dilute acetic acid solution);

c,d,e L-, M-, H-DEChN groups (fed a high-fat diet and low, medium, high doses, respectively, of DEChNs dispersions). Data are presented as mean ± standard error.

* *p* < 0.05 compared with the control group;

** *p* < 0.01 compared with the control group.

Compared with the control group, a decrease in the weights of the renal, epididymal, and mesenteric adipose tissues were observed in the H-DEChN and L-DEChN groups. The H-DEChN group showed a greater decrease than the L-DEChN group. The weights of the renal ATW of mice in the L-DEChN group and the H-DEChN group were 0.235 ± 0.068 and 0.146 ± 0.041 g, respectively, compared with 0.418 ± 0.148 g in the control group and 0.234 ± 0.116 g in the blank group. The weights of the epididymal adipose tissue of mice in the L-DEChN and H-DEChN groups were 0.621 ± 0.16 and 0.412 ± 0.098 g, respectively, compared with 0.819 ± 0.238 g in the control group and 0.695 ± 0.107 g in the blank group. The weights of the mesenteric adipose tissue of mice in the L-DEChN and H-DEChN groups were 0.476 ± 0.187 and 0.471 ± 0.097 g, respectively, compared with 0.728 ± 0.085 g in the control group and 0.616 ± 0.17 g in the blank group. The weights of the renal, epididymal, and mesenteric adipose tissues of mice in the M-DEChN group were 0.268 ± 0.075, 0.700 ± 0.159, and 0.594 ± 0.115 g, respectively. There was an obvious decrease in the weights of the collected adipose tissue compared with the control group, while there was no significant difference in the renal, epididymal, and mesenteric ATW/BW ratios of the M-DEChN group and the blank group. The results indicated that DEChNs could interfere with the absorption of dietary fat and cholesterol *in vivo*. This finding was likely a result of the unique ability of chitin to bind lipids and bile acids. The H-DEChN group (75 mg/kg·bw·d DEChNs) was more effective than the L-DEChN group (18.75 mg/kg·bw·d DEChNs) or the M-DEChN group (37.5 mg/kg·bw·d DEChNs) in decreasing the accumulation of lipids in tissue and preventing the development of obese mice.

The serum lipid concentrations of mice were measured, including TC, TG, HDL-C, and LDL-C. The results are shown in Table 3. After 21 days of experimental diets, the serum TC concentration of the mice fed the high-fat diet was higher than that of the mice fed a normal-fat diet. However, the serum TC and HDL-C concentrations of the mice in the H-DEChN group were lower than those of mice in the control group; the value of high density lipoprotein cholesterol (HDL-C) 1.658 mmol/L in the control group decreased to 0.678 mmol/L in the H-DEChN group, which is approximately a 2.44-fold decrease, indicating that while feeding the mice a high-fat diet normally resulted in a higher accumulation of lipids in the blood, DEChNs can suppress hyperlipidemia in mice that are fed high-fat diets.

**Table 3:** Serum lipid levels of mice

Groups	Blank^a^	Control^b^	L-DEChNs^c^	M-DEChNs^d^	H-DEChNs^e^
TC (mmol/L)	3.562 ± 0.786	3.910 ± 0.596	3.321 ± 0.589^*^	3.801 ± 0.883^*^	3.429 ± 0.507^*^
TG (mmol/L)	1.389 ± 0.430	1.247 ± 0.559	1.385 ± 0.514	1.311 ± 0.660	0.709 ± 0.234
HDL (mmol/L)	1.665 ± 0.406	1.658 ± 0.830	0.842 ± 0.346^**^	0.702 ± 0.176^**^	0.678 ± 0.263^*^
HDL/TC	0.468 ± 0.225	0.424 ± 0.133	0.253 ± 0.134	0.185 ± 0.084^**^	0.198 ± 0.124
LDL (mmol/L)	0.614 ± 0.198	0.697 ± 0.210	0.812 ± 0.271	0.849 ± 0.313	0.728 ± 0.166

TC, total cholesterol; TG, triacylglycerol; HDL-C, high density lipoprotein cholesterol; LDL-C, low density lipoprotein cholesterol

a Blank group (fed a normal diet and saline solution);

b control group (fed a high-fat diet and dilute acetic acid solution);

c,d,e L-, M-, H-DEChN groups (fed a high-fat diet and low, medium, high doses of DEChN dispersions, respectively). Data are presented as mean ± standard error.

* *p* < 0.05 compared with the control group;

** *p* < 0.01 compared with the control group.

### Liver histological examination

**Fig. 3:**
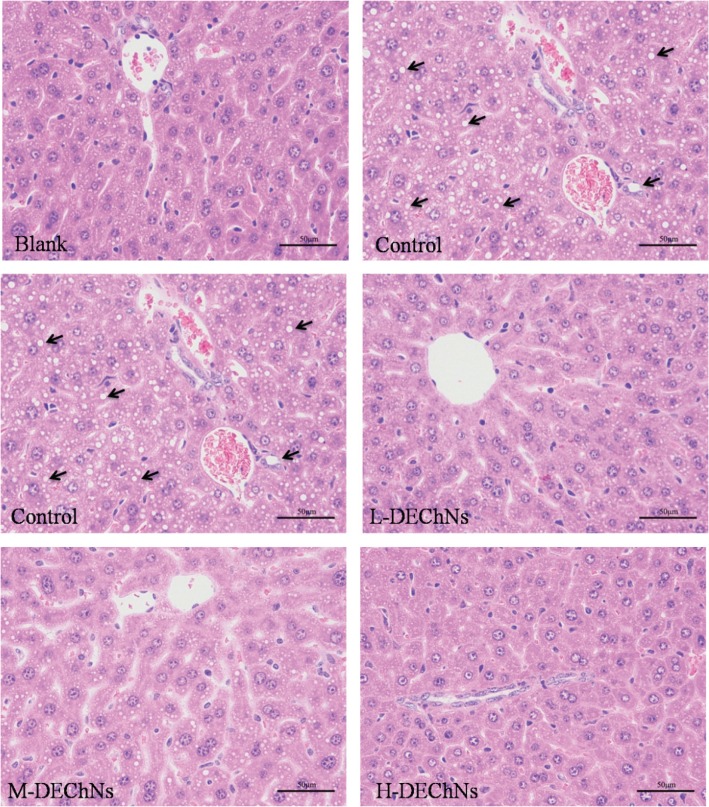
Histological examination of liver tissues from mice in the blank group (fed a normal diet and saline solution), control group (fed a high-fat diet and dilute acetic acid solution), and the L-, M-, H-DEChN groups (fed a high-fat diet and low, medium, high doses of DEChN dispersions, respectively). The arrows point out the fat vacuole in the cytoplasm.

The livers from the mice were subjected to histopathological examination, and the results are shown in Fig. 3. At the end of the experiment, the livers of the mice from the control group had become swollen, oily, and orange. By comparison, the livers from the blank and DEChN groups were normal and dull red in color. As shown in Fig. 3, there were no evident fat vacuoles in the cytoplasm from the blank group, while hepatocytes from mice in the control group had accumulated a large number of fat vacuoles. The results indicated that feeding the mice a high-fat diet resulted in a higher accumulation of lipids in the livers of mice, thereby leading to a severe fatty liver. Histopathological examination of hepatocytes from mice in the DEChN groups revealed that livers had a small number of droplet accumulation compared with those in the control group. In addition, there was no evidence of fat vacuoles in the cytoplasm among the DEChN groups and the livers of the DEChN groups appeared normal, that is, similar to the blank group. These results suggested that a small dosage of DEChN dispersions was effective in decreasing the accumulation of lipids in the liver, thus preventing the development of a fatty liver. The histopathological examination of the liver was consistent with the data collected for BW gain, ATW gain, and serum lipid levels, thereby proving that DEChNs possesses effective hypolipidemic activity.

### Optimum dosage of deacetylated a-chitin nanofiber/nanowhisker mixtures

Azuma et al. recently proposed that an oral administration of surface-deacetylated chitin nanofibers (SDCNFs) decreased the diet-induced increase in serum TC, chylomicron, very-low-density lipoprotein, phospholipid levels, and suppressed vacuolar degeneration and accumulation of lipid droplets in liver tissue (17). They also suggested that administration of SDCNFs did not affect BW change or organ weights. However, our study showed that DEChNs significantly reduced the BW and ATW gains in mice when fed a high-fat diet. The difference may be attributed to the dosage of DEChN dispersions. In the present study, the dosage was under precise control and the DEChNs were intragastrically administered.

The present study suggests that the BW and ATW gains of the mice in the DEChN groups were significantly lower than those of the control group and changed dramatically in the H-DEChN group. On the contrary, the changes of the weight ratio between tissue and body were observed to be slight. For the H-DEChN group, the suppression of BW gain and the reduction of adipose tissue accumulation were the most obvious. The drastic changes may indicate that mice fed excessive DEChNs exhibited a pathological growth state. Therefore, in consideration of the efficacy of ATW loss and ensuring healthy growth of mice as mentioned above, a dosage of 37.5 mg/kg·bw·d DEChNs (the M-DEChN dose), in the medium dosage range, was recommended and proposed in the present study.

At the recommended optimum dosage, DEChNs can facilitate weight loss and reduce accumulation of fat in diet-induced obese mice and significantly reduce the rise of liver lipid levels in mice when fed a high-fat diet. Meantime, at this dosage, the DEChNs reduced the plasma lipid levels, the serum TC, and TG concentration as well. These values were lower than those of mice fed the high-fat diet and dilute acetic acid solution, indicating that DEChNs can effectively prevent hypercholesterolemia when consuming a high-fat diet. The most probable explanation for this is the binding of lipids to the DEChNs, thereby reducing the absorption of dietary fat and cholesterol *in vivo*.

Chitosan, with an average molecular weight of 650 kDa or greater, was approved by the Ministry of Health, Labour and Welfare (Japan) in 1997 as a specific healthy functional food, and the approved oral dosage of chitosan was 0.5–3 g per day in humans (22). In particular, doses between 2 and 5 g/kg were used to evaluate the hypolipidemic effects of chitosan. In this study, the best dosage of DEChNs for optimal hypolipidemic effects was 37.5 mg/kg·bw·d, which is much lower than the recommended dosage of chitosan. Thus, we conclude that the hypolipidemic effects of DEChNs are more efficient than those of chitosan.

### Possible hypolipidemic mechanism of deacetylated a-chitin nanofiber/nanowhisker mixtures

According to previous reports, chitosan, a derivative of chitin, can reduce BW and ATW gains in mice that are fed high-fat diets (22). The hypolipidemic mechanism of chitosan was investigated in male Sprague-Dawley rats and the results suggested that chitosan reduced the absorption of dietary fat and cholesterol *in vivo*, thereby effectively reducing the occurrence of hypercholesterolemia in rats (23). There have been many studies that have attempted to explain the mechanisms of chitosan in inhibiting hyperlipidemia. A classical perspective, which is widely accepted, is that chitosan binds lipids in the gastrointestinal tract, which are then excreted in the feces (23, 24). DEChNs have a similar molecular structure to chitosan but can be identified by their nanofibrillar morphology, lower amino groups, and increased crystallinity. However, there have not been many research studies that have examined the use of DEChNs in supporting the hypolipidemic activities of mice, as well as the underlying mechanisms of how the DEChNs inhibit hyperlipidemia.

Our zeta-potential detection revealed that even though the DEChNs possess lower amino groups (DDA values approximately 0.25–0.35) than chitosan (DDA values normally >0.70), they still possess high cationic surface charges under acidic conditions. That is to say, chitin, as well as chitosan, may have the unique ability to bind lipids and bile acids. Moreover, the nanofibrillar morphology and high crystallinity might be the other advantages. In detail, the C2-amino groups on the surface of the DEChNs were cationized in gastric juice (pH 1–2), which gave the DEChNs a high zeta-potential of +30–70 mV in the stomach. Positively charging the DEChNs allowed for the attraction and accumulation of anions, such as large amounts of lipids, cholesterol, and bile acid, to the DEChNs via electrostatic attraction. It is noteworthy to mention that the DEChNs do not dissolve at a molecular level but disperse at a nanofibril level in the stomach. Therefore, the DEChNs adsorb to the surface of an oil droplet, decreasing its accessibility to digestive enzymes, thereby decreasing its digestion. Moreover, the DEChNs could act as an emulsifier, affecting the emulsification and adsorption of lipids because DEChNs and oil could contribute to a large accumulation through hydrophobic interaction and nano-effects. Therefore, the binding of lipids and the emulsification could be the main cause for the hypolipidemic action of DEChNs. When the protonated DEChNs enter the intestine (pH 7–7.5), they lose the positive charge then flocculate and precipitate along with the lipids, cholesterol, and bile acid. Thereafter, these precipitates are trapped in the intestine and eventually excreted.

## Conclusions

The present study examined the following effects of DEChNs: 1) reducing accumulation of adipose tissues, 2) preventing increases in plasma in mice that are fed a long-term high-fat diet, and 3) guarding against the symptoms of hypercholesterolemia and fatty liver in mice. Further studies are needed to examine the pharmacokinetics and biodegradability of DEChNs. Finally, the TC and lipid levels of the liver, serum, and liver enzymes and receptors regulating the lipid metabolism were not examined in this study. Therefore, these areas also need further examination.
